# Verbal Autopsy of Maternal Deaths in Two Districts of Pakistan—Filling Information Gaps

**DOI:** 10.3329/jhpn.v27i2.3329

**Published:** 2009-04

**Authors:** Sadiqua N. Jafarey, Talat Rizvi, Marge Koblinsky, Nazo Kureshy

**Affiliations:** ^1^ National Committee for Maternal and Neonatal Health, 36C, 14^th^ Street, Khayaban-Shamsheer, Phase 5, DHA, Karachi, Pakistan; ^2^ Department of Obstetric and Gynecology, Ziauddin University, Karachi, Pakistan; ^3^ Public Health Sciences Division, ICDDR,B, GPO Box 128, Dhaka 1000, Bangladesh (present address: John Snow Inc., 1616 Ft Myer Drive, Arlington, VA 22205, USA); ^4^ Child Survival and Health Grants Program, United States Agency for International Development, Ronald Regan Building, 1300 Pennsylvania, NW, Washington, DC, USA

**Keywords:** Causes of death, Maternal mortality, Risk factors, Socioeconomic factors, Verbal autopsy, Pakistan

## Abstract

In Pakistan, the vital registration system is weak, and population-based data on the maternal mortality ratio are limited. This study was carried out to collect information on maternal deaths from different existing sources during the current year—2007 (prospective) and the past two years—2005 and 2006—(retrospective), identify gaps in information, and critically analyze maternal deaths at the community and health-facility levels in two districts in Pakistan. The verbal autopsy questionnaire was administered to households where a maternal death had occurred. No single source had complete data on maternal deaths. Risk factors identified among 128 deceased women were low socioeconomic status, illiteracy, low-earning jobs, parity, and bad obstetric history. These were similar to the findings of earlier studies. Half of the women did seek antenatal care, 34% having made more than four visits. Of the 104 women who died during or after delivery, 38% had delivered in a private facility and 18% in a government facility. The quality of services in both private and public sectors was inadequate. Sixty-nine percent of deaths occurred in the postpartum period, and 51% took place within 24 hours of delivery. The study identified gaps in reporting of maternal deaths and also provided profile of the dead women and the causes of death.

## INTRODUCTION

Pakistan ranks the sixth most populous country in the world, with a population of 153.4 million in 2005. The population growth rate of 1.9% remains high despite a decline and is a significant factor in negating improvement in the socioeconomic and health indicators of Pakistan (1). The various health indicators, including maternal mortality ratio (MMR), continue to be poor and are a matter of concern.

However, the reporting system of health-related issues is weak. This lack of accurate information is one of the causes of inadequate planning.

Estimates by different sources show an MMR ranging from 350 to 500 per 100,000 livebirths (2). Data on maternal morbidity and stillbirths are even more scarce. All the above gaps need to be filled to help policy-makers make effective strategies with the resources available.

One method of addressing the gaps concerning causes of maternal deaths is by conducting a verbal autopsy of pregnancy-related deaths. The verbal autopsy tools and their validation have been refined over the last few years. A number of studies based on thousands of deaths have been reported from China and parts of Africa (3-12). In India, verbal autopsies have been used widely for ascertaining the causes of death among children and used effectively to influence health policy, programmes, monitoring, and evaluation.

In Pakistan, due to lack of vital registration and poor certification for cause of death, verbal autopsies offer a promising solution. Verbal autopsies have been used on a small scale earlier (13,14), and since 2003, the National Programme for Family Planning and Primary Health Care has expanded their use (14). The recently-concluded Pakistan Demographic and Health Survey (PDHS) also measured maternal mortality for the first time, based on interviews about deaths of females aged 12-49 years in about 96,000 households during the previous three years (15).

This study was undertaken to determine (a) the medical and socioeconomic causes of maternal deaths in a defined population and (b) accuracy and completeness of various sources of reporting of maternal deaths.

## MATERIALS AND METHODS

### Site selection

The two districts of Sindh selected were Sukkur district, which comprises four subdistricts with a population of 11,41,844 (urban 36.2%, rural 63.8%), and Malir district that includes three towns with a population of 10,41,029 (urban 67.2%, rural 32.8%). The criterion for selection of the two districts was high coverage for primary healthcare (PHC) and family planning by Lady Health Workers (LHWs) in their catchment populations. Each LHW covers a population of 1,000 or 150-200 households in the community and keeps a detailed record of every household, including information of all births and deaths. Death of any female aged 15-49 years reported by an LHW is verified to be a maternal death or not by the Assistant District Coordinator (ADC) of the programme who is a physician. It was, therefore, assumed that the data generated by the LHWs would be complete and a major source for identifying maternal deaths in the community.

### Development of verbal autopsy form

The National Committee for Maternal and Neonatal Health (NCMNH) developed the verbal autopsy questionnaire after reviewing verbal autopsy forms of the World Health Organization (WHO) and those used in projects in Chennai and Matlab and by the Population Council of Pakistan. The precoded form, used by the Population Council for a Safe Motherhood Study which included verbal autopsy in a district of Punjab and had already been validated, was modified slightly by omitting repetitive questions and questions with details of symptoms. This was used after pretesting in a non-research area. The questionnaire covered the following topics: household characteristics, background characteristics of women and family, reproductive history, healthcare and healthcare-seeking behaviour, birth-preparedness, referral, and delays.

The task of field work was assigned to the team of Community Health Sciences (CHS) Department of the Ziauddin University. This team has conducted many surveys in rural areas of Sindh for the university and for other international organizations, such as United Nations Children's Fund (UNICEF) and WHO. The field team comprised four female and three male workers who had completed at least 10-12 years of schooling. They were given a four-day training on information gathering, filling forms, and ways of proceeding in the field for initial identification of maternal deaths. They also pretested the verbal autopsy form.

Based on the available sources of information, the field teams with two senior members of the CHS department sought information on pregnancy-related deaths (deaths during pregnancy or within 42 days post-delivery) during the current year, 2007 (prospective) and the past two years (2005 and 2006—retrospective) from the following six sources: monthly reports of LHWs; health management information system (HMIS) available in the office of Executive District Officer (EDO)-Health, records of public-sector hospitals, records of private hospitals, graveyards, and union councils.

In addition to the above six sources of information, a survey was conducted in all the four subdistricts of Sukkur with almost complete coverage of the population of the four subdistricts. Two subdistricts were mainly urban and two largely rural. For field work, each subdistrict was divided into urban and rural areas. For the rural areas, a list of all villages was prepared while the urban areas were divided into blocks of approximately 50-100 households each, using landmarks.

The field workers accompanied by a member of the CHS Department of the Ziauddin University visited each village and block in the subdistrict and collected information on all deaths of females aged 15-49 years from key informants (*Imams* of mosques, school teachers, Councillors, and shop-keepers). Once the death of a female was identified, the female field worker visited the house seeking information on whether the death was related to pregnancy or not. If it was pregnancy-related death, the female field worker filled the detailed verbal autopsy form by interviewing persons who were present with the deceased at the time of her death. Wherever necessary, this information was supplemented with information given by other relatives and neighbours. In about a quarter of the cases, the NCMNH team also accompanied the field team.

In Malir district, however, the information collected on pregnancy-related deaths was available only through the records of LHWs. Attempts were made to get information from the union council and hospital records without results. Private hospitals were especially very secretive. Therefore, only two rounds of data collection were done. In the first round, a list was prepared of houses in the community where a pregnancy-related death was reported by the LHW and was verified as a maternal death by the Assistant District Coordinator of the LHW programme. In the second round, a team comprising the local LHW and her supervisors and one member of NCMNH visited these houses. Three such teams covered the district area and conducted verbal autopsy interviews. This team was different from the field team in Sukkur.

Three physician-members of the NCMNH reviewed the filled-in verbal autopsy tools collected from the field separately and assigned a cause of death. If there were agreement as to the cause of death by all three or two of the three members, this was taken as final. In the case of a disagreement between the members, the Principal Investigator resolved it. This was done in 20 (16%) cases. Results were analyzed using the SPSS software (version 11.0).

Causes of maternal deaths were categorized as: direct, indirect, co-incidental, and undetermined (co-incidental and accidental deaths are terms used interchangeably). While these latter deaths do not, in fact, conform to the WHO definition of maternal deaths, we have included them here. Eighty-four (36+48) deaths were recorded in Sukkur.

The causes of death were further classified as immediate, underlying, and associated. An attempt was also made to determine levels of delay according to the three-delay model in which the first delay is in deciding to seek professional care, the second delay is identifying and reaching an appropriate health facility, and the third delay is receiving adequate and appropriate treatment at the facility. Results presented in the study are obtained from three sources, i.e. records of LHWs, public and private hospitals, and the survey of Sukkur district.

### Data analysis

Data on maternal deaths were analyzed from the perspective of: (a) distal determinants, such as sociocultural and economic factors, including education, employment of women and husbands, and family status in the community; (b) intermediate determinants in terms of health and reproductive status of women and healthcare-seeking behaviour; (c) risk factors in terms of illnesses, including anaemia, past obstetric history, birth-preparedness, and family's knowledge/perceptions of risk factors; and (d) services factors in terms of types of services availed during pregnancy and in emergency, healthcare providers, referral routes, and services provided at hospitals. The three delays leading to death were also identified.

## RESULTS

### Sukkur district

Of the six sources tapped, three, i.e. LHWs, public hospitals, and private hospitals, provided information on maternal deaths. Information from the HMIS was limited to outpatients only. The records of the union council and graveyards did not identify any maternal deaths.

The records of LHWs showed 36 maternal deaths while public and private hospitals had records of 98 cases. None of the hospital cases, however, had complete addresses and could not, therefore, be traced. The survey carried out later identified 48 deaths, which were not in the LHW records. Thus, 84 maternal deaths were recorded in Sukkur during 2005-2007.

### Malir district

In Malir district, information on 44 cases was obtained from the records of LHWs. The other sources had no record of maternal deaths, although the families identified the hospitals nearby where the deceased were taken and where they had died. These were all private hospitals, including one tertiary hospital but when contacted, they informed that they did not have any maternal deaths in their hospitals. An NGO Health and Nutrition Development Society (HANDS) in Malir referred 36 women with complications to the Jinnah Postgraduate Medical Centre (JPMC), a government tertiary hospital, about 10 km away. Records of the JPMC showed that only 16 of those women reached the hospital, and all of them survived. Information on the remaining 20 cases was not available.

**Fig. 1.**
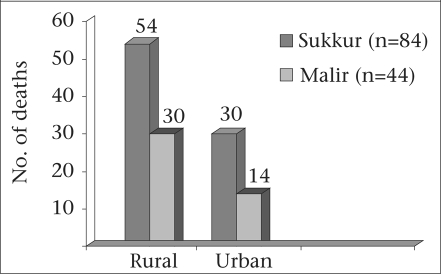
Maternal deaths by districts and rural/urban distribution (n=128), 2005-2007

This study is based on 128 maternal deaths identified through verbal autopsies from the two districts during 2005-2007. The urban-rural distribution is seen in Figure 1. Five of the deaths were classified as co-incidental. Four of these were due to suicide or domestic violence and the fifth due to anaphylactic shock following injection at a private clinic.

Table 1 shows the characteristics of the houses where the patients lived. In the rural areas, more than half (52%) of them lived in mud/brick and mud-huts with no sanitation facilities. The majority (59%) obtained water from home sources.

**Table 1.** Percentage distribution of deceased women (n=128) by housing characteristics, according to district and rural/urban distribution, 2005-2007

Characteristics	Sukkur (n=84)			Malir (n=44)			Grand total (n=128)	
	Rural	Urban	Total	Rural	Urban	Total	No.	%
Type of household								
Brick	10	19	29	22	10	32	61	48
Brick and mud	20	4	24	6	4	10	34	26
Mud-hut	26	5	31	2	-	2	33	26
Ownership of house								
Own	51	19	70	23	7	30	100	78
Joint ownership	2	2	4	5	-	5	9	7
Rented	-	5	5	1	6	7	12	9
Relative's house	1	4	5	1	1	2	7	6
Source of drinking-water								
In-home tap-water	15	16	31	11	6	17	48	38
Communal tap-water	2	6	8	8	5	13	21	16
In-home hand-pump/well	23	3	26	1	-	1	27	21
Communal hand-pump/well	14	5	19	10	3	13	32	25
Type of toilet								
Flush with septic tank	7	9	16	7	5	12	28	22
Latrine with open drain	19	18	37	17	9	26	63	49
Pit-latrine	3	-	3	4	-	4	7	5
Field	25	3	28	2	-	2	30	24
Electricity								
Yes	47	30	77	30	14	44	121	95
No	7	-	7	-	-	-	7	7

### Education and source of income

Eighty-seven (68%) women and 61 (48%) husbands had never attended school. The number of women with no education was more than double in rural areas compared to urban areas (69% vs 31%).

Ninety-eight (77%) urban women were housewives and were not doing any work for wages while 28 (22%) husbands had no employment at the time of the woman's death. In the rural areas, 83 (65%) husbands had low-earning jobs, such as labourers, street-vendors, or farmers. The husband of one woman only was a professional—an engineer.

### Reproductive and health status of women

Although all the 128 women were married, two were separating during the course of the index pregnancy. One hundred and three (80%) women were aged 20-39 years, 43 (35%) had more than five livebirths, and 12 (9%) had more than eight live children. One had died in her 16^th^ pregnancy. Twenty-two (17%) were primigravidas (Table 2).

**Table 2.** Percentage distribution of deceased women (n=128) by age, parity, inter-pregnancy interval, past obstetric and medical history according to district and rural/urban distribution, 2005-2007

Characteristics	Sukkur (n=84)			Malir (n=44)			Grand total (n=128)	
	Rural	Urban	Total	Rural	Urban	Total	No.	%
Age (years)								
<20	6	1	7	2	-	2	9	7
20-29	20	14	34	15	6	21	55	43
30-39	16	14	30	11	7	18	48	37
>39	12	1	13	2	1	3	16	13
Parity								
0	7	8	15	6	1	7	22	17
1-4	27	14	41	17	5	22	63	49
5-8	13	4	17	7	7	14	31	25
>8	7	4	11	-	1	1	12	9
Inter-pregnancy interval								
Primi/0	4	9	13	7	2	9	22	17
<24 months	14	8	22	5	2	7	29	23
24-36 months	24	10	34	8	6	14	48	37
>36 months	5	2	7	7	5	12	19	15
Do not know	4	-	4	1	-	1	5	4
Missing information	4	-	4	-	-	-	5	4
Past obstetric history								
Stillbirths	4	3	7	4	2	6	13	10
Spontaneous/induced abortions	11	3	14	6	3	9	23	18
Anaemia								
Yes	19	11	30	8	7	15	45	35
No	10	8	18	14	11	25	43	34
Do not know	8	4	12	3	7	04	16	12
Missing information∗	2	22	24	-	-	-	24	19
Medical illnesses (other than anaemia)								
Yes	10	8	18	15	11	26	44	34
No	11	12	23	14	3	17	40	32
Do not know	8	8	16	1	-	1	17	13
Missing information∗	2	25	27	-	-	-	27	21
Infections								
Hepatitis	6	2	8	2	1	3	11	9
Tuberculosis	1	-	1	1	2	3	4	3

∗Missing information for medical illness and anaemia during the index pregnancy is mainly from the survey record by the field team at two rural subdistricts of Sukkur

Twenty-nine (23%) women had the index pregnancy within 24 months of the last pregnancy, and 36 (28%) had a bad obstetric history as indicated by previous abortions or stillbirths. One woman had had four previous abortions.

Forty-five (35%) women were anaemic, of whom 12% were severely anaemic. During the index pregnancy, 44 (34%) women had medical illnesses, such as hypertension, jaundice, renal disease, and/or chest infections. The infective conditions mentioned were mainly hepatitis and tuberculosis.

### Antenatal healthcare and healthcare-seeking behaviour

During the index pregnancy, of the 73 (57%) deceased women who went for antenatal check-ups, 10 visited once, 18 visited 2-3 times, and 44 had more than four visits. In fact, 26 (36%) women had visited a healthcare provider during the week in which she died. Most (91%) women in Malir district, which is mainly urbanized, had made at least one antenatal visit. Attendance was lower in Sukkur (39%), the lowest (26%) being in the rural areas of Sukkur. Fifty-one (70%) women, who had made antenatal visits, attended private clinics or maternity homes while 22 (30%) had visited government health facilities (Table 3).

**Table 3.** Percentage distribution of deceased women (n=128) by number of antenatal visits, place, and last visit according to district and rural/urban distribution, 2005-2007

Characteristics	Sukkur (n=84)			Malir (n=44)			Grand Total (n=128)	
	Rural	Urban	Total	Rural	Urban	Total	No.	%
Antenatal visit								
Yes	11	22	33 (39%)	27	13	40 (91%)	73	57
No	39	7	46 (55%)	2	1	3 (7 %)	49	38
Do not know	4	1	5 (6 %)	1	-	1 (2 %)	6	5
Number of visits								
0	39	7	46	2	1	3	49	38
1	2	2	4	3	3	6	10	8
2-3	3	3	6	10	2	12	18	14
4 and above	6	17	23	14	7	21	44	34
Do not know	4	1	5	1	1	2	7	6
Total	54	30	84	30	14	44	128	
Place of ANC visit (n=73)								
BHU/RHC	-	-	-	4	-	4	4	5
Government hospital	3	7	10	3	5	8	18	25
Private clinics	8	15	23	20	8	28	51	70
Last antenatal visit before death								
Within a week	5	8	13	8	5	13	26
Within 2 weeks	4	8	13	7	3	10	23
Within a month	2	5	8	9	4	13	21	36
More than a month	-	1	1	3	1	4	3	
Total	11	22		27	13	40	73		

ANC=Antenatal care; BHU=Basic health unit; RHC=Rural health centre

Four of them tried to have the pregnancy terminated. In three cases, termination was attempted by the Lady Health Visitor (LHVs), in two of whom it failed and pregnancy continued while in one woman it was terminated with complications, i.e. visceral injury leading to her death. In one case, the physician declined to terminate the pregnancy due to the risk involved as she had had a previous caesarean section. The remaining three women died later due to puerperal sepsis, postpartum haemorrhage, and eclampsia.

### Birth-preparedness and knowledge of family regarding risk factors

In terms of birth-preparedness and complication-readiness, 52 (41%) women had decided themselves or with their husbands to have the delivery at a health facility. In 35 (27%) cases, the family decided to have the delivery at home.

Seventy-nine (62%) families had made arrangements for transport in the case of an emergency. One woman was taken to a tertiary-care hospital in a bus since no other transport was available in the village.

All had arranged for some money either from savings, selling household items/farm animals, or borrowed from the community. The respondents could not specify the exact amount of money spent since most of them were women, and men had dealt with the finances. Those who went to a hospital estimated an average of Rs 10,000 (US$ 160) in an emergency situation, with additional Rs 1,000-10,000 for buying supplies and drugs.

In response to the awareness regarding safe pregnancy and delivery, none of the family members interviewed was able to narrate three danger signs or risk factors. Fifty-eight (46%) were able to state only one danger sign/risk factor. The symptoms mentioned were bleeding (24%), cessation of foetal movement (11%), oedema (7%), and vomiting, palpitation, and general weakness by the rest.

### Place and type of delivery

Of 104 women who died during or after delivery, 44 (42%) had labour/delivery at home (husband's, parent's, or *dai's*/LHV's home), 39 (38%) in private hospitals, and 19 (18%) in government facilities while 2% died on the way to the hospital (Table 4).

**Table 4.** Percentage distribution of deceased women (n=104) by place of labour/delivery according to background characteristics of women and birth outcome, 2005-2007

Characteristics	Home/dai's/LHV's home	Government hospitals/TH	Private MH and hospitals	On the way	Total
Age (years)					
<20	1	1	4	-	6
20-29	23	6	15	-	44
30-39	18	7	13	1	39
>39	2	5	7	1	15
Parity					
0	2	5	7	1	15
1-4	27	7	16	-	50
5-8	10	4	12	1	27
>8	5	3	4	-	12
Education					
No	33	14	22	2	71
Primary	9	3	9	-	21
Secondary	2	2	5	-	9
College	-	-	3	-	3
Residence					
Rural	29	11	25	2	67
Urban	15	8	14	-	37
ANC visits					
None	20	5	8	-	33
1	3	1	-	1	5
2-3	8	1	8	1	18
>3	7	11	21	-	39
Do not know	6	1	2	-	9
Birth outcome					
Livebirth	34	11	27	-	72
Stillbirth	4	6	6	-	16
Died undelivered	6	2	6	2	16
Total	44	19	39	2	104

ANC=Antenatal care; DNK=Do not know; LHV=Lady Health Visitor; MH=Maternity home; TH=Taluka (subdistrict) hospital

Of 88 postpartum deaths, 64 (72.5 %) had normal vaginal deliveries (NVDs) while 20 (23%) had caesarean section and 4 (3.5%) had instrumental delivery.

### Stage of pregnancy when death occurred

Twenty-four (19%) women died during pregnancy, i.e. before labour started, 16 (12%) during labour, and 88 (69%) after delivery. The most critical postpartum period was the first 24 hours after delivery, with 45 women (43%) dying during this period and 33% within four hours of delivery.

### Category/cause of death

The number of direct maternal deaths was 91 (71%), indirect 19 (15%), and co-incidental 5 (4%), including four cases of suicide and/or alleged poisoning. In 13 (10%) cases, no cause could be determined (Table 5).

**Table 5.** Percentage distribution of deceased women (n=128) by category of death, delays according to district and rural/urban distribution, 2005-2007

Characteristics	Sukkur			Malir			Grand total (n=128)
	Rural (n=54)	Urban (n=30)	Total (n=84)	Rural (n=30)	Urban (n=14)	Total (n=44)	
Category of deaths							
Direct	35	24	59	22	10	32	91
Indirect	8	3	11	5	3	8	19
Co-incidental	3	1	4	1	-	1	5
Undetermined	8	2	10	2	1	3	13
Delay							
1st	12	9	21	9	6	15	36
2nd	5	6	11	1	-	1	12
3rd	13	11	24	10	5	15	39
Combination	9	3	12	2	3	5	17
None/ND	15	1	16	8	-	8	24

ND=Not determined

The first delay was identified in 36 (29%) women and the third delay in 39 (30%) women. In 23 (19%) cases, there was a combination of delays. The second delay alone was identified in 12 cases in both rural and remote areas. In 24 (21%) cases, no delay was detected or determined.

**Fig. 2.**
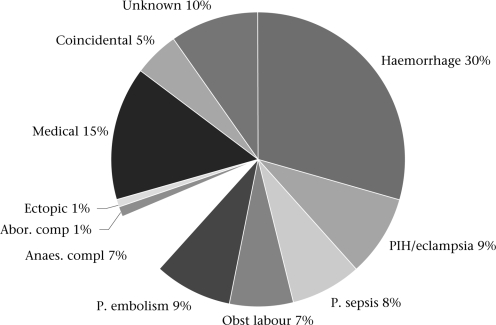
Causes of death (n=128), Sukkur and Malir districts, 2005-2007

The major causes of deaths identified are shown in Figure 2. Of the 88 postpartum deaths, 45 (51%) occurred during the first 24 hours of delivery and 30 (34%) as a result of postpartum haemorrhage.

Abor. comp=Abortion-related complication; Anaes. compl=Anaesthesia-associated complication; Obst=Obstructed labour; P. embolism=Pulmonary embolism; P. sepsis=Puerperal sepsis

Fifteen of 20 caesarean sections carried out were performed at private hospitals and five at government hospitals. Seven women who died due to intra-operative or immediate post-operative complications were those who had surgery in private hospitals

### Place of death

Eighty-two (64%) women died in health facilities (private and public). Thirty-three died at home and 13 on the way to hospital (Table 6).

**Table 6.** Percentage distribution of deceased women (n=128) by category of death according to age, parity, residence, and healthcare-seeking care, and place of death, 2005-2007

Characteristics	Direct (n=91)	Indirect (n=19)	Co-incidental (n=5)	Unknown (n=13)	Total (n=128)	
					No.	%
Age (years)						
<20	7	2	-	-	9	7
20-29	38	7	2	8	55	43
30-39	34	8	3	3	48	37.5
>39	12	2	-	2	16	12.5
Parity						
0	15	3	1	3	22	17
1-4	47	8	3	5	63	49
5-8	18	7	1	5	31	25
>8	11	1	-	-	12	9
Residence						
Rural	57	13	4	10	84	66
Urban	34	6	1	3	44	34
Antenatal care						
0	33	6	3	7	49	38
1	8	1	-	1	10	8
2-3	12	4	1	1	18	14
>3	36	6	-	3	45	35
Do not know	2	2	1	1	06	5
Place of death						
Home∗	20	4	1	8	33	26
Government	21	6	2	3	32	25
Private	29	4	2	1	36	28
Taluka Hospital	10	4	-	-	14	11
On the way	11	1	-	1	13	10
Total	91 (71%)	19 (15%)	5 (4%)	13 (10%)	128	100

∗Husband's home, parent's home, friend's or LHV's home; LHV=Lady Health Visitor

### Outcome of newborn

Of the eighty-eight women who died postpartum, 72 had livebirths, 14 had stillbirths, and in two cases, information was missing. Of the 72 livebirths, 50 were alive at the time of interview while 19 had died (Table 7 and 8).

**Table 7.** Status of liveborns at the time of interview (2005-2007)

Status	Number	Percentage
Still alive	50	70
Died	19	26
DNK	3	4

DNK=Do not know

**Table 8.** Age at time of death of 19 newborns, 2005-2007

Age	No. (n=19)
Birth–7 days (early NNDs)	10
>7-28 days (late NNDs)	7
1 month–1 year	2

NNDs=Neonatal deaths

## DISCUSSION

The present study provided information on two areas, viz. the medical and socioeconomic causes of maternal deaths in a defined population and the accuracy and completeness of information on maternal deaths from various sources. This study, done by a systematic in-depth analysis in two districts of Sindh, Pakistan, has revealed facts and issues relating to maternal deaths some of which were known.

Two-third of the deaths occurred in rural areas. The general profile of the women who died was comparable with earlier studies which showed that the majority of them belonged to the low socioeconomic class and were not educated (13). Half of the women were aged more than 30 years, one-third had more than four children, and 12 (9%) had more than eight children.

High parity is a reflection of lack of family planning as shown by a persistently low contraceptive prevalence rate (CPR) (15).

Anaemia during pregnancy remains a matter of concern in Pakistan, although the National Nutrition Survey reported a decline from 88% in 1965 to 36% in 2001-2002 (16). In the present study, the prevalence of anaemia either alone or in combination with another illness was the same (35%) as in the National Nutrition Survey of 2001-2002.

The persistently high prevalence of anaemia underscores the importance of maternal nutrition. The national programme for iron-fortified wheat needs to be expanded and its community-based activities strengthened. The programme was initiated in 2000 and is still very sporadic.

The tasks of LHWs include providing iron tablets to women of reproductive age in the community. However, it was difficult to determine whether the high prevalence of anaemia was due to non-compliance of women in taking tablets, omission in tablets being provided to them, non-absorption of iron, or whether there was a supply problem at the LHW level or above.

Moreover, many cases of jaundice/hepatitis were seen in the families during the field visits, indicating a high prevalence of the infection. This high prevalence of hepatitis is supported by the community surveys in the same areas. In a study conducted among first time blood donors in Karachi, the prevalence of HBV was found to be higher in Sindhi or minor language-speaking people than in those speaking Urdu (17). Recognizing the high prevalence of hepatitis, a National Programme for Hepatitis Prevention and Control was launched in 2005 to substantially decrease the prevalence, morbidity, and mortality due to viral hepatitis in the general population. 

The women with cause of death attributed to suicide or possible homicide died under suspicious circumstances. Although the reported number of cases of domestic violence in the present study was not high, there is an increasing concern about violence towards women, especially during pregnancy. Results of a study on domestic violence before and during pregnancy in Karachi showed that 23% of pregnant women were physically abused during the index pregnancy (18). 

In contrast to the widespread assumption that most women who die a maternal death do not go for antenatal care, our study found that 73 (57%) of the deceased women did go for antenatal check-ups, and 34% had made more than four visits. A third had sought antenatal care in the week they died. It could not be determined whether the visits were routine or whether the deceased went for a problem. Seventy percent chose private clinics or maternity homes, and the major reason for the choice was the assumption that the quality of services provided was good.

Similarly, the private hospitals and maternity homes were preferred over the government health facilities for delivery, even in the rural areas because of their anticipated better quality of care. More than 50% of the families, however, reported that the quality of services was not as good as expected, and 70% of the families felt that there had been mismanagement by the healthcare providers.

There is generally a trend towards having professional help at delivery. This is not only true in Pakistan: data from household surveys of developing countries showed that professional help at birth rose from 40% in 1992 to 50% by 2000, with a noticeable rise in births by doctors (19). In the present study, 62% of the women who died had a skilled birth attendant during labour/delivery.

The majority (64%) of the mothers died in a tertiary hospital—public or private—contrary to the belief that most women die at home.

In women who developed complications at home, there were delays in reaching a tertiary hospital, despite the maximum distance to the hospitals, being not more than 10-15 km. This was due to healthcare-seeking path starting with a traditional birth attendant/local *dai* who called in a local ‘doctor’. In the majority of the cases, these ‘doctors’ did not advise immediate referral. If the condition of the woman did not improve, she was taken to a nearby health facility in which appropriate personnel and supplies were not always available. Only when her condition deteriorated was she referred to the tertiary hospital. This delay in referral has earlier been reported in Sindh (20).

When a complication occurred in a health facility (public or private), the woman was generally referred directly to a government tertiary hospital. The narrative data indicate that there were delays in receiving immediate and appropriate care even after reaching the tertiary hospital. Unfortunately, this information cannot be validated as the condition of the patient on admission (moribund or otherwise) and the time elapsed between admission and death were not available in the hospital records. Nevertheless, the quality of care, especially emergency obstetric care (EmOC), is a matter of concern as shown by two other surveys—one in Sindh and one in Punjab (21-22).

Twenty-eight percent of women died in private hospitals, and the commonest cause was postpartum haemorrhage. The presence of anaemia worsens the prognosis in the cases of postpartum haemorrhage. It was satisfying to note that, in all the 34 cases where blood was required, the family members donated it. This is contrary to the general belief that families are hesitant in donating blood. Moreover, in all the cases where blood was arranged, it was, in fact, transfused to the patient.

The first delay and the third delay were identified in the majority of the cases. The second delay emerged as a problem only in 9% of the cases from the rural areas. This is because of the wider availability of ambulances provided by Edhi, a social welfare organization, and Suzuki pick-ups on rent.

It is widely recognized that it is difficult to ascertain a cause of death reliably through verbal autopsies. Nevertheless, the majority of deaths were determined to be due to direct causes. Haemorrhage (mostly postpartum haemorrhage) was by far the most common cause of death (30%). A large number of these women came from the rural areas of Sukkur where access to EmOC is difficult, and they did not reach the facility on time. In 12 (9%) women, the deaths were sudden, and the symptoms were suggestive of embolism. The medical causes contributed to 15% of the deaths and included hepatitis and cardiac disease.

These causes of death are consistent with reports from Pakistan—both community and hospital (13,23,24)—and from other developing countries (25), except for the low (1%) proportion of deaths due to unsafe abortion. Hospital studies in Pakistan showed that 11-12% of all deaths were due to complications of abortion—both spontaneous and induced (23). Globally, unsafe abortion is among the five most common causes of maternal deaths, accounting for 13% of the total number of deaths. Nearly 90% of these occur in developing countries, nearly half of which are in Southeast Asia (26). The low number of women seeking abortion in this study might have been due to under-reporting—deliberate or otherwise—since induced abortion is perceived to be illegal and has stigma attached to it. In fact, abortion is legal to save the life of the woman or for ‘necessary treatment’.

The one woman who had died as a result of complication of abortion received post-abortion care by an LHV, resulting in visceral injury.

The fact that 68% of rural women who died, died of a direct cause reflects the lack of accessibility to high-quality emergency obstetric services. Nineteen of the 38 deaths due to postpartum haemorrhage were among those who had delivered at home by *dais*.

The Maternal Mortality Survey 2001 in Bangladesh reported similar findings. The most common cause of death was haemorrhage (29%). However, the proportion of deaths due to eclampsia was high (24%). The explanation given was that, “since eclampsia exerts its effects slowly”, the women might have been referred to an EOC facility which might have been unable to solve the problem (27).

All seven deaths attributed to anaesthesia-associated complications were among the 15 women who underwent caesarean sections in the private hospitals. This raises serious concerns about the quality of care, especially the facilities available and the skills of anaesthetists. In the private sector, it was not possible to determine the type of anaesthesia given and the competency of the anaesthetist.

In developing countries, nearly half of all maternal deaths occur during labour or in the immediate postpartum period (18). In Bangladesh, over 60% of deaths occurred after delivery, one in 10 during labour, and one in five before labour (27). In our study too, 69% died in the postpartum period, nearly half of them dying within 24 hours of delivery. Even then, postpartum care is the most-neglected component in maternal and infant care. Women who deliver at home are left without monitoring during this critical period, and most women who deliver in health facilities are sent home within 24 hours of birth. Care during this period is crucial to prevent both maternal and neonatal deaths and needs attention within the maternal and neonatal health programmes. Yet, postpartum care is available to only 22% of women in Pakistan, and the quality of care is highly debatable.

The amount of money spent (Rs 10,000-20,000) on facility care is equivalent to one month's income for most families. Fourteen families had sold valuables, including farm animals.

Seventy percent of the babies born alive to women who died were still alive at the time of the interview. These children were being looked after by members of the extended family, including aunts and grandmothers. This is in contrast to a study carried out in Matlab, Bangladesh, during the 1980s, in which it was found that, of 125 children born to mothers who died, three-fourths had died within 12 months (28).

One very important fact that emerged from this study was that family members were not very aware of risk factors/danger signs of pregnancy and childbirth. The most commonly-cited alarming symptom was bleeding, followed by cessation of foetal movement. This is in contrast to the findings of the needs assessment survey of PAIMAN, a USAID- funded MNH project in Pakistan, which showed that 53% and 29% of women in the community were aware of three danger signs during pregnancy and the postpartum period respectively (29).

Health education to women and families is one of the responsibilities of LHWs. When the LHWs were questioned about this deficiency, the large majority, i.e. almost 80% of them, attributed it to additional tasks being expected of them, allowing them very little time to deal with maternal health in general, and primary healthcare and family planning in particular. Local NGOs can play a major role in creating awareness both in community and among healthcare providers as is being done by the Health & Nutrition Development Society (HANDS), an NGO in Malir.

Six sources were tapped for providing information on maternal deaths. The results were not very encouraging. The LHWs' and hospital records were the only sources from where information on maternal deaths was obtained though incomplete. The other sources, i.e. graveyards, union councils, and the HMIS of the Ministry of Health (MoH), did not report on deaths in a manner that allowed maternal deaths to be indentified.

This necessitated a survey of subdistricts of Sukkur. It was expected that reporting of maternal deaths by LHWs would be complete, this being one of the tasks of LHWs. However, 48 deaths, i.e. 57% of all maternal deaths, that were identified later through the survey of the four subdistricts in Sukkur were missing in the records of LHWs. This deficiency in reporting and verification of maternal deaths was also found in the National Programme of LHWs in which verbal autopsies of maternal deaths by district managers have been included since 2001 (14).

After devolution of district administration in 2002, the system of vital registration and information management at union councils has been strengthened through management training. A death certificate issued either by union councils or a hospital is required for burial. Although records of female deaths were available at the union councils, there was no information as to whether the deaths were maternal or not. The field team visited the houses of the deceased women to determine whether any of these deaths was maternal in nature. None was identified.

Recently, the Karachi City Government has signed an agreement with the National Data Base Registration Authority (NADRA) to develop a computerized system of births, deaths, marriages, and divorce records. This Civil Registration Management System (CRMS) will cover all union councils of the metropolis, including Malir district. The NCMNH has had meetings with concerned authorities to include relevant information in the death forms that would identify maternal deaths.

No maternal deaths were identified from the graveyards. The caretakers of the majority of graveyards are not educated and, thus, cannot document or keep records. Moreover, they are not even aware that, in registered graveyards, a death certificate is required before burial.

The study has brought out the reluctance of private health facilities, especially in Malir, to share their data and accept that maternal deaths have occurred. This is unfortunate and needs to be redressed.

### What needs to be done

In addition to addressing the issues of poverty, under- and malnutrition, low literacy among both males and females, empowerment of women, and gender inequity, the conclusions drawn from this study prompt recommendations in two specific areas: (a) causes and determinants of maternal deaths and (b) reporting/record-keeping of maternal deaths.

### Addressing causes and determinants of maternal deaths

- **Audit maternal deaths in both hospital and community settings:** The faculty at the hospitals needs to analyze all maternal deaths occurring at the facility to educate junior staff and students (medical and midwifery) not only on the medical causes but also on the sociocultural factors leading to these deaths and how these could be prevented.
  The Government should give authority to a responsible body to audit deaths at home and health facilities, especially small maternity homes, when and if necessary. The NCMNH and Society of Obstetricians and Gynaecologists of Pakistan have offered to take up this responsibility jointly.
- **Increase emphasis on intrapartum and post-partum care:** To prevent postpartum haemorrhage, active management of the third stage of labour should be offered to all women delivered by SBAs. For deliveries by TBAs, buccal/sublingual Misoprostol should be promoted to prevent and manage postpartum haemorrhage. This could be administered by the TBA or taken by the woman herself.
  Labour and the first 24-48 hours postpartum period should be monitored very closely. If the woman delivers in the health facility, she should be monitored during this crucial period. If she is sent home earlier, arrangements should be made to ensure monitoring. If she delivers at home, this responsibility is of the attendant at delivery who should be trained to recognize complications early.
- **Strengthen the role of LHWs for safe motherhood, including family planning:** The role of LHWs for safe motherhood and family planning should be strengthened as was designed initially. The low CPR reported in the PDHS indicates the need for this emphasis.
- **Increase the accessibility and availability of family planning:** Family-planning services should be made available at the doorstep, especially in rural areas. All available sources, including LHWs and Family Welfare Workers of the Ministry of Population Welfare, should be used.
- **Increase skilled attendance and im proved quality of training of SBAs:** Competency-based training of all SBAs, including doctors and community midwives, as part of the national maternal and child-health strategy, during the next five years, should be ascertained. The skills of the EmONC team which includes an obstetrician, an anaesthetist, a neonatologist, and a midwife should be maintained by repeated hands-on training. All anaesthetists should be trained to give spinal anaesthesia.
  For the community midwives, a regulatory mechanism should be developed so that they function effectively.
  Improved teaching skills of teachers should be ensured.
- **Increase availability and accessibility of 24/7 EmONC services and improvement of qual ity of care:** This component is included in the continuum of care model of the PAIMAN project in Sukkur, which is one of the 10 sites of the project. It needs to be strengthened in all the districts.
- **Increase awareness of danger signs among the community:** In addition to the LHWs, NGOs should play a major role in creating awareness in the community regarding early recognition of danger signs and the appropriate facility to seek help as is being done in Malir district.
- **Strengthen the programme to prevent and control hepatitis:** The National Programme for Prevention and Control of Hepatitis, launched in 2005, needs to be strengthened and implemented effectively. Screening of all pregnant women and immunization of all adolescent females, especially in high-prevalence areas, should be ascertained.

### Strengthening the system of reporting/record-keeping of maternal deaths

The strengthening is required in both HMIS and the National Data Base Registration Authority.

The ongoing verbal autopsy being carried out by the LHW programme needs further streamlining. Records (including addresses) of hospital admissions should be complete and accurate. All birth attendants in both public and private sectors should be made aware of the importance of reporting maternal deaths.

Most importantly, dissemination of the findings of the study to the policy-makers, planners, professionals, and the community at appropriate forums for sensitization and action should be ensured.

[Addendum: Since the submission of this paper, the final report of the Pakistan Demographic and Health Survey 2006-2007 has been released. The national maternal mortality ratio is reported to be 276 per 100,000 livebirths.]

## ACKNOWLEDGEMENTS

The information and views presented in this article are solely those of the authors and do not necessarily represent the views or the positions of the U.S. Agency for International Development or the U.S. Government.
